# Anti-Excitotoxic Effects of N-Butylidenephthalide Revealed by Chemically Insulted Purkinje Progenitor Cells Derived from SCA3 iPSCs

**DOI:** 10.3390/ijms23031391

**Published:** 2022-01-26

**Authors:** Hsin-Han Yang, I-Tsang Chiang, Jen-Wei Liu, Jeanne Hsieh, Jui-Hao Lee, Huai-En Lu, Hwa-Sung Tso, Yu-Chen Deng, Jo-Chi Kao, Jhen-Rong Wu, Horng-Jyh Harn, Tzyy-Wen Chiou

**Affiliations:** 1 Department of Life Science and Graduate Institute of Biotechnology, National Dong Hwa University, Hualien 974, Taiwan; 810413102@gms.ndhu.edu.tw (H.-H.Y.); 610513102@gms.ndhu.edu.tw (I.-T.C.); 410613012@gms.ndhu.edu.tw (H.-S.T.); curosakihisoka@gmail.com (Y.-C.D.); kaojochi@gmail.com (J.-C.K.); 410713008@gms.ndhu.edu.tw (J.-R.W.); 2 Everfront Biotech Inc., New Taipei City 221, Taiwan; coldwee@efbiotech.com (J.-W.L.); jeannehsieh@gmail.com (J.H.); juihaolee@efbiotech.com (J.-H.L.); 3 Bioresource Collection and Research Center, Food Industry Research and Development Institute, Hsinchu 300, Taiwan; hel@firdi.org.tw; 4 Bioinnovation Center, Buddhist Tzu Chi Foundation, Hualien 970, Taiwan; 5 Department of Pathology, Buddhist Tzu Chi General Hospital and Tzu Chi University, Hualien 970, Taiwan

**Keywords:** n-butylidenephthalide, SCA3, ATXN3, iPSCs, Purkinje progenitor, quinolinic acid

## Abstract

Spinocerebellar ataxia type 3 (SCA3) is characterized by the over-repetitive CAG codon in the ataxin-3 gene (*ATXN3*), which encodes the mutant ATXN3 protein. The pathological defects of SCA3 such as the impaired aggresomes, autophagy, and the proteasome have been reported previously. To date, no effective treatment is available for SCA3 disease. This study aimed to study anti-excitotoxic effects of n-butylidenephthalide by chemically insulted Purkinje progenitor cells derived from SCA3 iPSCs. We successfully generated Purkinje progenitor cells (PPs) from SCA3 patient-derived iPSCs. The PPs, expressing both neural and Purkinje progenitor’s markers, were acquired after 35 days of differentiation. In comparison with the PPs derived from control iPSCs, SCA3 iPSCs-derived PPs were more sensitive to the excitotoxicity induced by quinolinic acid (QA). The observations of QA-treated SCA3 PPs showing neural degeneration including neurite shrinkage and cell number decrease could be used to quickly and efficiently identify drug candidates. Given that the QA-induced neural cell death of SCA3 PPs was established, the activity of calpain in SCA3 PPs was revealed. Furthermore, the expression of cleaved poly (ADP-ribose) polymerase 1 (PARP1), a marker of apoptotic pathway, and the accumulation of ATXN3 proteolytic fragments were observed. When SCA3 PPs were treated with n-butylidenephthalide (n-BP), upregulated expression of calpain 2 and concurrent decreased level of calpastatin could be reversed, and the overall calpain activity was accordingly suppressed. Such findings reveal that n-BP could not only inhibit the cleavage of ATXN3 but also protect the QA-induced excitotoxicity from the Purkinje progenitor loss.

## 1. Introduction

Spinocerebellar ataxia type 3 (SCA3) is a polyglutamine neurodegenerative disorder in which patients exhibit a wide range of motor dysfunctions such as impaired balance, uncontrolled muscle tensing (dystonia), and stiffness (spasticity) in the early onset and progressive limb incoordination including peripheral neuropathy and muscle twitches (fasciculations) [1]. To date, SCA3 is the most common SCA subtype in Japan, Taiwan, China, and Portugal, and there remains no effective treatment [2]. The over-repetitive CAG codons in exon 10 of the *ATXN3* gene, which encode abnormal ataxin-3 (ATXN3) aggregation, play a pivotal role in the neuropathogenesis of SCA3. In healthy individuals, the CAG repeats ranged from 12 to 44, whereas the CAG repeats ranged from 56 to 91 in SCA3 patients [3]. The abnormal CAG repeats in exon 10 of the ATXN3 gene lead to ATXN3 protein misfolding and accumulation of the abnormal ATXN3 protein, which has been demonstrated to be the hallmark of the SCA3 pathogenesis [4]. The protein quality control mechanism including the ubiquitin–proteasome pathway, molecular chaperones, and autophagy were also dysregulated in SCA3 [5]. The affected protein homeostasis further induces the misfolding of nuclear proteins, DNA damage, mitochondria dysfunction, and the induction of intracellular Ca2^+^ release, which result in neuron degeneration [4].

The function of ATXN3 protein is related to the ubiquitin–proteasome pathway [6]. ATXN3 substrates or interacting proteins are reported to be associated with DNA repair factor recruitment, cell cycle arrest, and DNA repair [5], and the dysfunctional mutant ATXN3 may lead to the accumulation of DNA damage. Enhanced DNA damage has been observed in SCA3 patients [7] and SCA3 transgenic mice [8], which provide another molecular mechanism of SCA3 pathogenesis. Additionally, glial cells, such as astrocytes, oligodendrocytes, and microglia cells, are activated in SCA3 patients [9,10,11]. Inhibition of NF-κB in astrocytes can delay neurodegeneration in Drosophila SCA3 containing an expanded polyglutamine (polyQ) repeats (SCA3^polyQ78^) model [12], suggesting the regulation of glial cells might be important to SCA3 disease progression. Drugs that target neurotransmitter modulation, ion transport inhibition, histone deacetylase (HDAC) inhibition, and autophagy enhancement have been tested in SCA3 patients through clinical trials [13].

The accumulation of mutant ATXN3 in neuronal nuclei is a characteristic of SCA3 neuropathology [14]. In neuronal cells, ATXN3 locates in the cytoplasm under basal conditions [15]. However, under cellular stresses, nuclear localization of ATXN3 can be observed [16]. Forcing mutant ATXN3 into the nucleus induces earlier death in SCA3 mice [17], which implies that the distribution of ATXN3 is important to SCA3 disease progression. The over-repetitive polyQ encoded by CAG in C-terminal expansions in the ATXN3 protein is further cleaved by calpain in the proteolytic process [18]. Subsequently, the abnormal, proteolytic fragments of ATXN3 formed pathological aggregates in the neuronal nucleus and were observed in the brains of SCA3 patients, which are involved in the molecular pathogenesis of SCA [18]. It was reported that after intracerebroventricular injection of quinolinic acid (QA), the triggered calcium influx further activated the calcium-dependent calpain, resulting in the increased production of ATXN3 proteolytic fragments [19,20]. Association of calpain-dependent proteolytic cleavage with truncated C-terminal ATXN3 was critical to the formation of ATXN3 proteolytic fragments during the progression of SCA3 [18,21]. The calpain system in the central nervous system contains calpain 1, calpain 2, and calpastatin. Calpain 1 and calpain 2 exert calcium-dependent proteolytic cleavage, and calpain 2 has higher cleavage efficiency on ATXN3 than calpain 1 [4,22]. The function of calpastatin is to inhibit the activity of these calpains [23]. Additionally, inhibition of calpain activity by calpastatin in the mouse brain prevents mutant ATXN3 proteolysis, nuclear localization, and aggregation [22].

In our previous works, we demonstrated that n-butylidenephthalide (n-BP), a small molecule drug, could exhibit anti-aggregation and autophagic modulation and could largely improve the motor coordination and activity in the HEK293T-GFP-ATXN3-84Q cells and a transgenic SCA3 ATXN3-84Q animal model, respectively [24]. In addition, the decreased tryptophan 2, 3-dioxygenase (TDO2), and calpain activity were indicated to be the underlying mechanisms [20].

In the last decade, the development of induced pluripotent stem cells (iPSCs) provides relatively reliable models for studying human disease pathogenesis as well as platforms for drug discovery [25,26]. However, the differentiation and maturation of Purkinje cells from iPSCs usually take 70 to 90 days [27,28], which is unfavorable for developing drug screening platforms. To meet the needs, we aimed to investigate the feasibility of using QA-induced Purkinje progenitor cells (PPs) derived from patient iPSCs as a drug screening platform for SCA3 in this study. Furthermore, we examined the anti-excitotoxic effect of n-BP on the established in vitro human SCA3 PP model.

## 2. Results

### 2.1. The Generation and Characterization of PPs

In order to perform the Purkinje linage differentiation, iPSCs were dissociated, transferred to AggreWell dishes, and cultured with gfCDM containing bFGF for 10 days to form embryoid bodies (EBs). At day 11, the EBs were then transferred to laminin and coated cell culture dishes and cultured with gfCDM containing bFGF for another 25 days. After 35 days of Purkinje linage differentiation, ICC staining and flow cytometry were used to characterize the yielded PPs.

**Figure 1:**
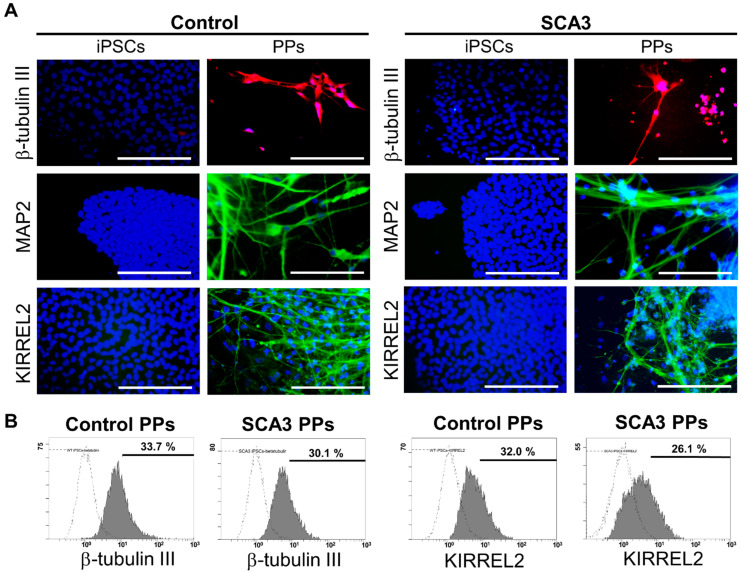
**The differentiation of control and SCA3 iPSCs into Purkinje progenitor cells (PPs).** The control and SCA3 iPSCs were differentiated with gfCDM as described in Materials and Methods. At 35 days of differentiation, both control and SCA3 iPSCs exhibited similar characteristics of PPs when compared with a previous study [29]; (**A**) morphological patterns of neural markers β-tubulin III and MAP2, and Purkinje lineage markers KIRREL2, as reflected by ICC staining. Scale bar = 100 μm; (**B**) the flow cytometry examination of neural marker β-tubulin III and Purkinje lineage marker KIRREL2 to determine the differentiation yield.

As shown in Figure 1A, after 35 days of differentiation, both the control and SCA3 iPSCs revealed neuron-like morphology and expressed neural markers such as β-tubulin III and MAP2. In addition, KIRREL2 was expressed in both control and SCA3 iPSCs-derived neuronal cells, as observed under fluorescence microscope (Figure 1A), indicating that both the control and SCA3 iPSCs were successfully differentiated into PPs after 35 days of differentiation. To evaluate the differentiation yield, flow cytometry analysis of neural and Purkinje cell-related markers was performed. As shown in Figure 1B, the culturing with gfCDM resulted in around one third of Purkinje progenitor differentiation (33.7% for control PPs vs. 30.1% for SCA3 PPs in neuronal linage marker β-tubulin III; 32.0% for control PPs vs. 26.1% for SCA3 PPs in Purkinje progenitor linage marker KIRREL2) on both control and SCA3 iPSCs.

### 2.2. Chemical-Insulted SCA3 PPs Represent the Pathological Phenotype of SCA3

In order to investigate whether the SCA3 PPs can exhibit pathological characteristics in the presence of QA, the analysis of neurite loss, apoptotic status as indicated by PARP1 expression, and the co-localization of expression of mutant ATXN3 were performed in SCA3 PPs after 35 days of differentiation.

**Figure 2:**
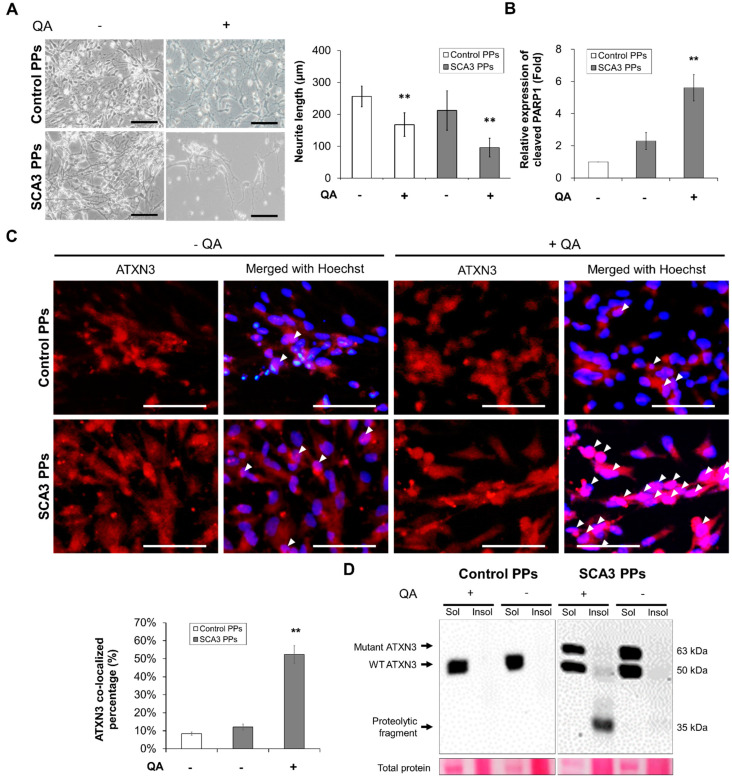
**The SCA3 PPs exhibited pathological characteristics after the treatment of QA.** The control and SCA3 PPs were treated with 1 μM of QA for 12 h. (**A**) The representative morphological observation and the quantification of neurite length analysis of control- and SCA3 PPs from three independent images. Scale bar = 100 μm. Data are presented as the means ± standard deviation; t(28) = 6.945, *p* < 0.01, for Control PPs vs. Control PPs with QA; t(28) = 14.16, *p* < 0.01, for Control PPs vs. SCA3 PPs with QA; (**B**) protein analysis of PARP1 in cell lysates of control and SCA3 PPs by immunoblots under the treatment of QA in the presence of variable concentrations of n-BP. Statistics refer to lane 1 to lane 3 of Figure 3D. Data are presented as the means ± standard deviation. t(4) = 5.876, *p* < 0.01, for SCA3 PPs vs. SCA3 PPs with QA; (**C**) the representative image of ATXN3 nucleus co-localization assessed by ICC assay. The co-localization is indicated using white arrowheads and the quantification data from three independent images are presented as the means ± standard deviation. t(4) = 13.74, *p* < 0.01, for SCA3 PPs vs. SCA3 PPs with QA; Scale bar = 100 μm; (**D**) protein analysis of wild-type, mutant ATXN3 and its proteolytic fragment in cell lysates of control and SCA3 PPs by immunoblots. Sol, soluble protein; Insol, insoluble protein form. **, *p* < 0.01.

**Figure 3:**
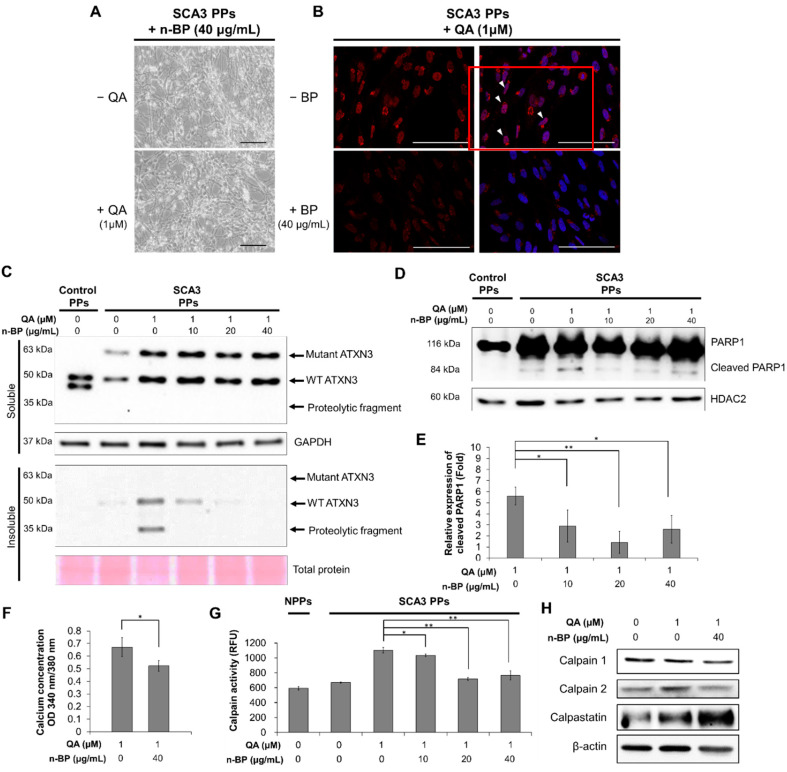
**The treatment of n-BP rescued the progression of QA-induced excitotoxicity in SCA3 PPs.** The control and/or SCA3 PPs were treated with or without QA (1 μM) in the presence of n-BP (0, 10, 20, and 40 μg/mL) for 12 h. (**A**) Images of QA-treated SCA3 PPs in the presence of BP (40 μg/mL). (**B**) Confocal images of poly Q localization in BP (40 μg/mL)-treated SCA3 PPs in the presence of QA (1 um). When compared with Figure 2C, 40 μg/mL of n-BP prevented the amounts of colocalized polyQ in the cell nucleus of PPs. The distribution of polyQ in the nucleus is indicated by white arrowheads. Scale bar = 100 μm; (**C**) protein analysis of wild-type, mutant ATXN3 and its proteolytic fragment in cell lysates of control and SCA3 PPs, as assessed by immunoblots. Soluble, soluble protein; Insoluble, insoluble protein; (**D**,**E**) representative immunoblot of control and cleaved PARP1 in cell lysates of control and SCA3 PPs, as assessed by immunoblots. The HDAC2 were used as loading controls. The quantification from three independent images is presented as the means ± standard deviation. t(4) = 2.824, *p* < 0.05, for lane 1 vs. lane 2; t(4) = 5.613, *p* < 0.01, for lane 1 vs. lane 3; t(4) = 3.522, *p* < 0.05, for lane 1 vs. lane 4; (**F**) the quantification result of calcium concentration in SCA3 PPs, as assessed by Fura-2 indicator. Cells were treated with QA (1 μM) in the presence of n-BP (0, 10, 20, and 40 μg/mL). Data are presented as the means ± standard deviation. t(4) = 2.95, *p* < 0.05; (**G**) the calpain activity in control and SCA3 PPs cell lysates, as assessed by ELISA assay. The quantification results from three independent replicates are presented as the means ± standard deviation. t(4) = 3.27, *p* < 0.05, for lane 3 vs. lane 4; t(4) = 17.55, *p* < 0.01, for lane 3 vs. lane 5; t(4) = 9.019, *p* < 0.01, for lane 3 vs. lane 6. (**H**) Protein analysis of calpain 1, calpain 2 and calpastatin in cell lysates of SCA3 PPs, as assessed by immunoblots. Cells were treated with or without QA (1 μM) in the presence of n-BP (0, 10, 20, and 40 μg/mL). *, *p* < 0.05; **, *p* < 0.01.

As shown in Figure 2A, compared with the control PPs, the morphological changes including the shrinkage and the decreased neurite area of SCA3 PPs were observed after the treatment of QA. Additionally, the expression level of cleaved PARP1, a hallmark during apoptosis and necrosis, was also found higher in the SCA3 PPs than that in the control PPs (Figure 2B, statistics refer to lane 1 to lane 3 of Figure 3D).

The other feature of SCA3 is the nuclear translocation of the proteolytic fragments and the further aggregation of insoluble ATXN3, which is required for expressing the disease symptom. Hence, we analyzed the distribution of ATXN3 protein by immunofluorescent staining. As indicated by the white arrowheads in Figure 2C, fewer ATXN3 signals were detected in the cell nucleus of control PPs after the stimulation of QA. However, as stimulated by QA, lots of ATXN3 localized in cell nucleus were observed in the SCA3 PPs. Consistently, via immunoblot, the protein expression of insoluble ATXN3 was detected in the cell lysates of SCA3 PPs and was rarely found in those of control PPs (Figure 2D).

Collectively, our results demonstrate that the SCA3 iPSCs can successfully differentiate into PPs with higher proteolytic fragments after 35 days of differentiation and that they were more sensitive to the excitotoxicity induced by QA than control PPs.

### 2.3. Chemical-Insulted SCA3 PPs Represent the Pathological Phenotype of SCA3

Since the pivotal roles of calcium-dependent calpain have been demonstrated in previous studies [19], the calcium concentration and calpain activity in SCA3 PPs were examined. In addition, the drug n-BP, which has been found to reduce motor neuron degeneration in a transgenic SCA3 mouse model in our previous study [20], was used to investigate the neuroprotective effects on SCA3 PPs.

We first examined the effect of n-BP on ATXN3 co-localization. As shown in Figure 3A,B, we found that the pretreatment of BP at 40 ug/mL did not affect the neurite length in the non-QA or QA-treated SCA PPs. In addition, the treatment of BP inhibited the formation of ATXN3 co-localization (Figure 3B), as indicated through the polyQ staining, indicating that BP could inhibit ATXN3 co-localization without side effects. We further found that the ATXN3 proteolytic fragments observed in the insoluble protein fraction of QA-induced SCA3 PPs (Figure 2D) was abolished in the n-BP treatment group (Figure 3C). Moreover, the death of PPs, as reflected by the expression levels of cleaved PARP1, were found decreased in QA-induced SCA3 PPs in the presence of n-BP (Figure 3D,E). These results suggest that n-BP can inhibit the QA-induced excitotoxicity by reducing the production of proteolytic fragments to hinder SCA3 disease progression.

Since the activity of calpain is regulated by calcium, the calcium concentration was examined by intracellular calcium indicator Fura-2. As shown in Figure 3F, decreased calcium concentration was observed in the QA-induced SCA3 PPs in the presence of n-BP. In addition, decreased calpain activity and expression were found (Figure 3G), indicating the reduction in proteolytic activity. We further examined the calpain enzyme complex that associates with the ATNX3 proteolytic pathway. The calpain system is composed of calpain 1, calpain 2, and their inhibitor, calpastatin. We showed that the expression levels of calpain 1 were not affected by n-BP. However, downregulated calpain 2 and upregulated calpastatin were found (Figure 3H) to be correlated with the calpain activity analysis result (Figure 3G). These results indicate that n-BP regulated the expression levels of calpain 2 and calpastatin to reduce calpain activity, and subsequently limited proteolytic activity. It resulted in lowered production of ATXN3 proteolytic fragments, as revealed by the decreased ATXN3 proteolytic fragments shown in the insoluble fraction of Figure 3B.

## 3. Discussion

Our results reveal that QA treatment worsened the pathological phenotypes, compromised intracellular calcium stability, increased the calpain activity, and promoted neuronal degeneration in the SCA3 PPs. We demonstrated that the induction of QA successfully stimulated SCA3 PPs to display neuropathological characteristics. This established SCA3 PPs system provides a time-saving drug screening platform as compared with the methods for the generation of mature Purkinje neurons [27,28].

Generally, the excitotoxicity, which is triggered by the over-stimulated NMDA receptors, is critical in the neuronal death and the progression of neurodegenerative diseases [30,31]. In this study, we found that the NMDA stimulator QA induced greater neurotoxic impact on SCA3 PPs than control PPs. Excitotoxicity was reported to be critical in the progression of neurodegenerative diseases [30]. Figure 3F indicates that the calpain activity in control PPs and SCA3 PPs are quite similar before the induction of QA. The calpain activity of SCA3 PPs activated by QA triggered the production of mutant ATXN3 proteolytic fragments to induce cytotoxicity. The unobserved cell death (Figure 2A) and ATXN3 fragments (Figure 2D) in control PPs treated with QA implied that the calpain activity was not activated by QA in the control PPs group. This may provide an explanation for why a healthy person without the *ATXN3* gene mutation will not acquire the neuronal cytotoxicity, even under the daily stimulation of calcium influx.

Moreover, n-BP was found to ameliorate the neuropathological characteristics in SCA3 PPs. According to our results, n-BP exerted neural-protective effects under the concentrations ranging from 10 to 40 μg/mL. With obvious dose–response in the PPs system, 40 μg/mL of n-BP was used to analyze the effect on calpain activity, proteolytic fragment formation, and neural degeneration. When QA-insulted SCA3 PPs were treated with n-BP, upregulated expression of calpain 2 and concurrent decreased level of calpastatin could be reversed, and the overall calpain activity was accordingly suppressed. Such findings reveal that n-BP not only could inhibit the cleavage of ATXN3 but also could protect the QA-induced excitotoxicity from the Purkinje progenitor loss. Our studies suggest that QA-associated pathways greatly contribute to the neuropathological phenotypes, such as the accumulation ATXN3 aggregates and that these pathways can serve as drug targets for developing potential SCA3 therapeutics.

The disease pathological characteristics of SCA3 are attributed to the production and accumulation of ATXN3 proteolytic fragments. The increased calcium concentration may activate calpain 2 to produce ATXN3 proteolytic fragments as seeds for protein aggregate formation [18]. Additionally, the major intracellular protein degradation pathways, including autophagy and the ubiquitin–proteasome system [32], were inhibited in SCA3 [33,34,35]. It was reported that inhibition of calpain activity using calpain inhibitor calpeptin resulted in the protective effect against the over-stimulated excitotoxicity [22]. In this study, it is revealed that the amount of proteolytic ATXN3 proteolytic fragment was reduced by n-BP through the upregulation of calpastatin and the inhibition of calpain 2. Furthermore, it was reported that the inhibition of calpain activity resulted in the activation of autophagy pathway in a zebrafish model [36]. Our team revealed that the inhibitory calpain activity of n-BP could promote autophagic process in this excitotoxic PPs model and MJD84.2 SCA3 transgenic mouse model [24]. These findings affirm that both reduced ATXN3 proteolytic production and promoted protein degradation pathway contribute to the ATXN3 reduction in the cell nucleus and the insoluble protein fraction in QA-induced SCA3 PPs under n-BP treatment. 

In our previous report, n-BP reduced the expression of TDO2 and could further prevent the endogenous QA-induced over-excitation of Purkinje cells in a SCA3 animal model [20]. Herein, our data show that exogenous QA-induced excitotoxic effects in PPs were reduced by n-BP treatment. These findings indicate that n-BP not only regulated metabolic TDO2 protein but also mediated calcium influx. With n-BP treatment, the concentration of calcium in SCA3 iPSC-derived PPs was decreased. However, the detailed mechanism of modulating calpain activity and inhibiting the over-excited toxicity needs further investigation.

## 4. Materials and Methods

### 4.1. Cell Culture of Control and SCA3 iPSCs

The male SCA3 patient-derived iPSCs (TVGH-iPSC-SCA3-04) from Taipei Veterans General Hospital were kindly provided by Huai-En Lu, Bioresource Collection and Research Center (BCRC), Food Industry Research and Development Institute, Hsinchu, Taiwan. The characterization results of SCA3 patient-derived iPSCs have been published recently [37]. Normal iPSCs (NTUH-01-05), which were derived from healthy human subjects, were purchased from BCRC and used as the control iPSC in this study. Characterization and validation results of the Control iPSCs are disclosed on the BCRC website: https://catalog.bcrc.firdi.org.tw/ (21 June 2021). The cells were cultured according to the published paper and the guidance from BCRC [37]. Briefly, cells were cultured with mTeSR™1 (85850, Stemcell, Vancouver, BC, Canada) consisting of 1× penicillin/streptomycin (Simply) at 37 °C in a humidified atmosphere containing 5% CO_2_. The mTeSR™1 was replaced every 24 h in order to prevent the unexpected differentiation. The cells were passaged by using accutase (SCR005, Merck-Millipore, Burlington, MA, USA) and mechanical scraping since they reached 80% confluency.

### 4.2. Differentiation of Control and SCA3 iPSCs into PPs

The differentiation method was modified from a previous published method [31]. The iPSCs were treated with accutase for 2–5 min and harvested by scraping when they reached to 80% confluency. A total of 6000 dissociated iPSCs were then transferred to AggreWell 800 (Stemcell, BC, Canada) dishes and cultivated with gfCDM containing 20 ng/mL bFGF (233-FB, R&D, Minneapolis, MN, USA) for 10 days to form embryoid bodies (EBs). At day 11, the EBs were then transferred to laminin (5 μg/mL; 11243217001, Sigma, SL, USA) and poly-D-lysine (50 μg/mL; A-003-E, Sigma)-coated cell culture dishes and cultivated with gfCDM containing 20 ng/mL bFGF (233-FB/CF, R&D) for 25 days to form Purkinje progenitor cells. During the procedure of EB formation and Purkinje progenitor cells differentiation, the cell culture medium was replaced every other day.

### 4.3. Induction of Excitotoxicity on the PPs and the Treatment of n-BP

After 35 days of PPs differentiation, 1 μM of QA in the presence of n-BP (0, 10, 20, 40 μg /mL, dissolved in the DMSO as the 10% (*w/v*) n-BP/DMSO stock, ALFA, Turin, Italy) was introduced for 12 h to induce the excitotoxicity. After the induction, immunocytochemistry, flow cytometry, ELISA, and Western blotting analysis were performed to characterize the QA-induced PPs.

### 4.4. Immunocytochemistry (ICC) Staining

To characterize the differentiated PPs and the ATXN3 expression levels in PPs, ICC staining was performed. Cells seeded in cell-culture slides were fixed with 4% paraformaldehyde (Millipore, Burlington, MA, USA) for 10 min and washed with phosphate-buffered saline (PBS) for 3 times. The cells were then blocked and permeabilized with 1× PBST containing 0.01% Triton-100 and 1% BSA for 1 h. Cells were then incubated with primary antibodies at 4 °C overnight, followed by 1× PBST washing step for 3 times. The staining of fluorescence-conjugated secondary antibody was then applied for 1 h, followed by 1× PBST washing and 5 min Hoechst33342 (1:2000; H1399, Invitrogen, Waltham, MA, USA) staining steps. After the final 1× PBST washing steps, the slides were mounted and then observed under a fluorescence microscope (Olympus, IX70, Tokyo, Japan) and confocal microscopy (Zeiss LSM510, Oberkochen, Germany).

The primary and fluorescence-conjugated secondary antibodies used in this study were as follows: MAP2 (1:400; ab32454, Abcam, Cambridge, UK), β-tubulin III (1:300; sc5274, Santa Cruz, TX, USA), ATXN3 (1:1000; MAB5360, Millipore), KIRREL2 (1:1000; AF2564, R&D), anti-mouse Alexa594 (1:5000; A-11032, Invitrogen), anti-rabbit Alexa488 (1:5000; A-11008, Invitrogen), anti-sheep Alexa488 (1:5000; A-11015, Invitrogen), anti-goat Alexa594 (1:5000; A-11058, Invitrogen), anti-polyglutamine-expansion disease marker, polyQ (MAB1574, Merck Millipore).

### 4.5. Measurement of Neurite Length and Quantification of ATXN3 Co-Localization

For measuring neurite length, a stereological assessment was performed in this study. Briefly, the representative micrographs of culture area of control and SCA PPs treated with or without QA were obtained under a microscope (Olympus, IX70, Tokyo, Japan). The full cell images were then superimposed with images containing a frame and eight test lines. The cells and the intersection amounts of neurite and test lines were manually counted using a computer mouse and transferred to corresponding neurite length (**L**) by the means of equation

$$L=\frac{\pi d}{2}I$$

where **I** represent the amount of neurite test line intersections, and **d** represents the vertical distance between two test lines.

For the quantification of ATXN3 co-localization percentage, at least three independent fields of each group were obtained. Then, the highly expressed fluorescence in cell nuclei were counted as ATXN3 co-localization manually. The percentage of ATXN3 co-localization was then calculated by dividing the amounts of total cells.

### 4.6. Flow Cytometry

Flow cytometry was performed to determine the differentiation efficiency. The cells were treated with trypsin for 2–5 min and harvested, followed by 400× *g* centrifugation for 5 min. The supernatant was removed, and the cells were resuspended to 1 × 10^6^ cells/mL in ice cold DPBS, 0.1% BSA.

Primary antibody staining was then applied on the cells according to the manufacturer’s instructions. The washing steps were performed 3 times by centrifuging at 400× *g* for 5 min and resuspending them in ice-cold DPBS. After the washing steps, the cells were resuspended with diluted (according to the manufacturer’s instructions) fluorochrome-labeled secondary antibody in 0.1% BSA/DPBS for 1 h in dark, and followed by the 3 times washing steps mentioned above. Finally, all samples were acquired and analyzed on FC500 (Beckman, Brea, CA, USA).

The primary and fluorescence-conjugated secondary antibodies used in this study were as follows: KIRREL (1:100; AF2564, R&D), β-tubulin III (1:300; sc5274, Santa Cruz), anti-sheep Alexa488 (1:2000; A-11015, Invitrogen), anti-mouse Alexa555 (1:2000; A28180, Invitrogen), anti-rabbit Alexa488 (1:2000; A-11008, Invitrogen).

### 4.7. Enzyme-Linked Immunosorbent Assay (ELISA)

A calpain activity fluorometric assay ELISA kit (K240, Biovision, Milpitas, CA, USA) was used to determine the calpain activity of the PPs according to the manufacturer’s instructions. After neuronal differentiation, the accutase-treated cells were harvested and resuspended with 100 μL 1× RIPA lysis buffer (20–188, Millipore) for 20 min, then followed by 10,000× *g* centrifugation for 10 min. The concentration of total protein in the supernatants was determined by the Bradford method. For ELISA analysis, 50 μg of each total protein sample was resuspended in 85 μL 1× RIPA buffer, then added with 5 μL calpain substrate and 10 μL reaction buffer (10×). The 1 μL of active calpain was mixed with 84 μL 1× RIPA buffer, and the 85 μL of blank 1× RIPA buffer was also added with 5 μL calpain substrate and 10 μL reaction buffer (10×) to be regarded as the positive and negative control group. After 1 h reaction in dark, the relative fluorescence units (RFU) and concentration of these samples were read and measured by Multiskan GO (Thermo Fisher Scientific, Waltham, MA, USA).

### 4.8. Measurement of the Intracellular Calcium Concentration

In order to detect the intracellular calcium concentration, the PPs were cultured with medium containing RevitaCell supplement (1:100, A2644501, Gibco™, Thermo Fisher Scientific) and compound E (1:1000, 73952, Stemcell) in a Matrigel-coated 35 mm µ-Dish for three days. Then the cells were gently washed with 1 mL buffer solution (Ca2^+^ physiological buffer containing 10 mM glucose). After removing the buffer, the 0.5 mL Fura2-AM solution (F1221, Thermo Fisher Scientific) was applied on the cells for 40 min. Then the cells were washed by with 1 mL buffer solution, and followed by buffer incubating for 15 min. The cells were placed under an upright widefield epifluorescence microscope (ECLIPSE Ti2, Nikon, Tokyo, Japan) equipped with a 340/380 nm LED system (pE-340^fura^, CoolLED, Andover, UK).

### 4.9. Protein Extraction

Soluble and insoluble protein fractions were extracted to define the expression levels of the calpain system (calpain 1, calpain 2, and calpastatin) and ATXN3 (wild-type ATXN3, mutant ATXN3, and ATXN3 proteolytic fragment). The soluble protein was extracted by 1× RIPA buffer after cells were harvested. For the extraction of insoluble protein, the remaining precipitates of each sample after the removal of total protein supernatant were resuspended with 200 μL of 100% formic acid (27001, Sigma) for 16 h and followed by vacuum-drying until the formic acid was completely removed. Then, each sample was resuspended with 40 μL of urea buffer according to the previous method, and underwent sonication for 30 min to ensure the precipitates were completely dissolved. For the extraction of nuclear protein, a Nuclear Extraction Kit (2900, Millipore) was used according to the manufacturer’s protocol. The quantification of extracted protein was performed by Bradford assay (Bio-Rad, Hercules, CA, USA).

### 4.10. Western Blot Analysis

A total of 40 µg of protein was mixed with 0.1 M DTT (D1532, Invitrogen) and 4× LDS sample buffer (NP0007, Invitrogen) and heated at 95 °C for 5 min to prepare denatured-reducing protein. The denatured-reducing protein was applied to electrophoresis on a polyacrylamide gel and then transferred to polyvinyldene fluoride membranes. For blocking the nonspecific binding, 5% skim milk was used, and followed by the primary antibodies, incubating at 4 °C overnight. At 5 min of 1× PBST, washing steps were then performed for 3 times on membranes, and followed by 1 h incubation of HRP-conjugated secondary antibody at room temperature. After the final 3 times of 1× PBST washing steps, the chemiluminescence detection was performed, and the intensity of the signal was detected by an iBright CL1000 Imaging System (Thermo Scientific).

The primary antibodies used in this study were as follows: ATXN3 (1:1000; MAB5360, Millipore), calpain 1 (1:5000; GTX102340, GeneTex, Irvine, CA, USA), calpain 2 (1:5000; GTX111809, GeneTex), calpastatin (1:5000; GTX645540, GeneTex), PARP1 (1:1000, MAB600, R&D), GAPDH (1:10,000, ab8245, Abcam), β-actin (1:5000; MAB8929, R&D), and HDAC2 (1:5000; GTX109642, GeneTex).

### 4.11. Statistics

The statistical comparisons from at least three technical replicates and independent experiments were analyzed by SPSS 14. Data are summarized as the mean ± standard deviation. The *t* (degree of freedom) = the *t* statistic value, and the *p* value is provided in the figure legend. A *p* value < 0.05 was considered significant.

## 5. Conclusions

In conclusion, we generated PPs from SCA3 patient-derived iPSCs based on a 35-day differentiation process. The resulting PPs expressed both neural and Purkinje progenitor’s markers. The induction of QA stimulated the excitotoxicity and led to the presentation of pathological characteristics of SCA3 in SCA3 PPs. This in vitro iPSC PP model can serve as a platform to effectively screen potential therapeutics for SCA3 disease. Furthermore, in this study, the neural protective effect of n-BP was also demonstrated via the established SCA3 PP platform.

## Data Availability

Not applicable in this study.
